# Differences in Parents and Teachers’ Perceptions of Behavior Manifested by Gifted Children with ADHD Compared to Gifted Children without ADHD and Non-Gifted Children with ADHD Using the Conners 3 Scale

**DOI:** 10.3390/brainsci12111571

**Published:** 2022-11-18

**Authors:** Juliette François-Sévigny, Mathieu Pilon, Laurie-Anne Gauthier

**Affiliations:** Department of Psychology, Université de Sherbrooke, Longueuil Campus, Longueuil, QC J4K 0A8, Canada

**Keywords:** giftedness, gifted children, attention deficit/hyperactivity disorder, twice exceptionality, twice-exceptional children, behavioral rating scale, Conners 3, inattention, hyperactivity, misdiagnosis

## Abstract

The potential for the misdiagnosis of giftedness as attention deficit/hyperactivity disorder (ADHD) has been well documented, as has the clinical diagnostic profile of individuals with both giftedness and ADHD. This study aimed to examine parents’ and teachers’ responses to the Conners 3 behavioral rating scale of gifted students with ADHD compared to gifted students without ADHD and non-gifted students with ADHD. Ninety-two children aged 6 to 16 years were included in the study. On the basis of clinical assessments utilizing the K-SADS, the WISC-V, and other neurocognitive tests, the students were split into three groups: gifted/ADHD (*n* = 35), ADHD (*n* = 35), and gifted (*n* = 22). The results revealed that mothers’, fathers’, and teachers’ responses to the Conners 3 rating scale distinguished well between the gifted group and the other two groups, but not between the gifted/ADHD and ADHD groups. The learning difficulties observed by teachers was the most significant element that distinguished gifted/ADHD students from non-gifted ADHD students. Other results indicated that mothers and fathers reported more inattention problems in their gifted/ADHD children than teachers. Additionally, mothers tended to observe more learning and executive function problems in their gifted/ADHD children than teachers did. These findings highlight the importance of multiple informants complementing each other in the assessment process for ADHD in a gifted context to counteract the masking effect between giftedness and ADHD.

## 1. Introduction

Attention deficit/hyperactivity disorder (ADHD) is one of the most common neurodevelopmental disorders in school-aged children and is characterized by symptoms of inattention and hyperactivity–impulsivity that are present before age 12 and interfere with daily functioning in at least two settings (e.g., at home and at school) [1,2]. According to the DSM-5, there are three types of ADHD presentation [1]. The combined presentation is when there is the presence of both inattentive and hyperactive–impulsive symptoms; the inattentive presentation is when there is a stronger presence of inattentive symptoms; and the hyperactive–impulsive presentation is when there is a stronger presence of hyperactive–impulsive symptoms [1]. The prevalence of ADHD is estimated to be between 5% and 8% in school-aged children [2]. In addition to attention deficits, cognitive deficits, particularly executive dysfunction affecting working memory, inhibitory, control, and set shifting/flexibility, are central to ADHD [3,4,5]. Additionally, several studies have shown that children and adolescents with ADHD present significant social functioning impairment [6,7,8] that can be marked by rejection by peers and adults, less cooperation in group activities, and high involvement in interpersonal conflicts [8,9]. All functional impairments of ADHD have been linked to the development of low self-esteem in children and adolescents [10]. Furthermore, nearly 75% of children with ADHD are also affected by a comorbid disorder such as oppositional defiant disorder, conduct disorder, a learning disorder, a mood disorder, or an anxiety disorder [11,12,13]. Thus, the assessment of ADHD must be comprehensive and rigorous in order to identify comorbid conditions that may cloud a child’s clinical profile [14,15].

### 1.1. Giftedness and ADHD

Giftedness and ADHD can co-exist in children. This is known as “twice exceptionality” and is characterized by a potential for high performance in skills or creativity in one or more areas of activity coupled with the presence of a mental health or neurodevelopmental disorder such as ADHD [16]. The prevalence of ADHD in children with giftedness is similar to that of children without giftedness, ranging from 3% to 9% [14,17]. The results of some studies tend to show that gifted/ADHD children are a subgroup of the gifted population at increased risk for psychosocial and academic adjustment problems [17,18,19,20,21,22]. More specifically, in terms of neurocognitive functioning, gifted/ADHD children tend to have deficits in working memory and executive functions compared to gifted children without ADHD [18,19,20,22]. In terms of social and emotional functioning, gifted/ADHD children exhibit more relationship problems and symptoms of anxiety, depression, and opposition than their peers without ADHD [17,21]. In addition to reporting lower self-esteem and feelings of happiness than gifted children without ADHD, they maintain a more negative perception of their behaviors [23]. Academically, gifted/ADHD children are at greater risk of underachievement or academic failure compared to their peers without ADHD [21]. In this context, the early identification of ADHD is important in order to implement interventions that address the difficulties experienced by the child [24,25,26].

However, the accurate diagnosis of ADHD in the gifted population has proven to be very difficult [14]. One of the reasons is that the strengths and disabilities of gifted/ADHD children interact with each other in such a way that one can mask the other or they can mask each other [14]. This masking effect can take three possible forms [14]. The first involves ADHD masking the giftedness so that those around them only perceive the disabilities such as inappropriate behaviors or even suboptimal academic performance [14]. The second form is where the giftedness masks the ADHD. In this context, the child’s cognitive strengths compensate for their difficulties, which may be interpreted by others as a lack of effort or perseverance [16,27]. The third form is where giftedness and ADHD mask each other [16]. Thus, because neither of the child’s exceptionalities is identified, the child receives neither the supports necessary to minimize the impact of ADHD nor the intellectual stimulation necessary to develop his or her full intellectual potential (e.g., academic enrichment programs) [27].

In addition, gifted children may exhibit behaviors that look similar to the characteristics of ADHD, contributing to misdiagnosis. For example, a gifted child who is bored in class because they are not stimulated enough may become agitated, which may resemble some of the symptoms of ADHD. To this end, two studies have shown that pre-service teachers were more likely to assign a diagnosis of ADHD even when the alternative of giftedness was suggested to them [28,29]. Additionally, Rinn and Renolds [30] concluded in their study that the misdiagnosis of ADHD in gifted individuals could be due to a lack of awareness of the characteristics of giftedness, particularly regarding overexcitabilities such as psychomotor, sensory, and imaginational overexcitabilities. They add that these overexcitabilities are often interpreted as behaviors indicating the presence of ADHD. Still, too few studies have examined how behavioral manifestations of inattention or hyperactivity–impulsivity are expressed in gifted/ADHD children and how they are differently perceived by their parents and teachers. However, a deeper understanding of the differential display of ADHD in the gifted population would allow for better screening and limit diagnostic errors [31].

### 1.2. Standardized Behavioral Rating Scales in the ADHD Assessment Process among Gifted Children

In the assessment of ADHD in school-aged children, the use of observations provided by multiple informants, especially parents and teachers, is a particularly necessary practice considering that the diagnostic criteria for ADHD require impairment in at least two settings such as at home and at school [1]. In addition to providing a comprehensive assessment, multi-informant assessment is sensitive enough to detect variations in mental health, which supports accurate judgment-making by clinicians [32]. Guidelines for the evidence-based assessment of ADHD in children recommend the use of standardized behavioral rating scales with parents and teachers [33]. Standardized behavioral rating scales are intended to measure the degree to which a youth exhibits behavioral manifestations that represent symptoms of the disorder. However, since most standardized behavioral rating scales for ADHD do not include a subgroup of gifted students in their standardization sample, some caution is warranted regarding their use in this population.

Nevertheless, two studies have explored the symptoms of ADHD in gifted children using behavioral rating scales. First of all, in their study, Gomez and colleagues [34] observed differences in inattention and hyperactivity symptoms between gifted, gifted/ADHD, ADHD, and non-gifted children without ADHD using the SWAN behavioral rating scale [35]. Specifically, ADHD children presented more of these symptoms than other groups of children, followed by gifted/ADHD children. However, although in this study the diagnoses of ADHD were made by professionals, the behavioral rating scale for ADHD was completed only by the participants’ mothers. This is a limitation because, in clinical practice, using the parent as the only source of information is inadequate to determine whether the child meets the full diagnostic criteria for ADHD [36].

Furthermore, Wood [31] conducted the only study to date that examined the responses of parents and teachers of gifted students at risk of ADHD to the Conners 3 behavior rating scale. The results indicate that the parent and teacher ratings of these students were not significantly correlated and that there were no significant differences between them in terms of student ratings. However, the small sample size (*n* = 21), the lack of comparison groups, and the fact that the children did not have a formal diagnosis of ADHD limit the generalization of the results. Nevertheless, the author states the need for a study to compare between parent and teacher ratings of gifted children—with and without ADHD—as well as non-gifted children—with and without ADHD—to highlight differences in how ADHD symptoms are expressed by gifted children and in how these symptoms are perceived by parents and teachers. The author also claims that there is a need for further understanding of the use of behavioral rating scales, particularly the Conners 3, among gifted/ADHD students.

### 1.3. The Current Study

The primary objective is to examine parents and teachers’ responses to the Conners 3 behavioral rating scale for gifted/ADHD children compared to gifted students without ADHD (gifted children) and non-gifted ADHD children (ADHD children). Our first hypothesis was that parents and teachers would report more ADHD symptoms in ADHD children than in gifted children or in gifted/ADHD children (H1). Our second hypothesis was that symptoms of inattention, hyperactivity–impulsivity, and learning problems perceived by the parents and the teacher would significantly distinguish gifted children from gifted/ADHD and ADHD children (H2). The secondary goal is to explore the differences between mothers’, fathers’, and teachers’ responses of gifted children, gifted/ADHD children, and ADHD children to the Conners 3 rating scale. We hypothesized that mothers would rate all children more highly on the Conners 3 scale than fathers and teachers (H3). This study will provide us with a deeper understanding of the expression of ADHD in gifted children on the basis of the perceptions of several informants using one of the most widely used behavioral rating scales.

## 2. Materials and Methods

### 2.1. Participants and Procedures

Our clinical sample consisted of 92 drug-naïve children aged six to 16 years (*M_age_* = 9.85; *SD* = 2.51; 73.91% male), along with their parents (*n* = 174; *M_age_* = 42.71; *SD* = 4.98; 52.87% of the rating parents were mothers) and teachers (*n* = 92). While the gifted/ADHD group (71.43% male) and the ADHD group (71.43% male) were composed of 35 children, the gifted group was composed of 22 children (72.73% male). All were French-speaking participants. Data for all participants were collected from the Child and Adolescent Assessment and Intervention Clinic of the University of Sherbrooke in Longueuil (Quebec, Canada) and from one private clinic in Montreal (Quebec, Canada) from 2015 to 2021. The research project was approved by the Research Ethics Board of the Humanities and Social Sciences Department of the University of Sherbrooke. For the collection of the archival data, only data from records that included a signed consent form for data collection to be used in future research that met the study’s inclusion and exclusion criteria were extracted anonymously and entered into the database.

The criteria for inclusion in the gifted group were an IQ greater than or equal to 130 on the Full-Scale Intelligence Quotient (FSQI) or the General Aptitude Index (GAI) of the Wechsler Intelligence Scale for Children Fifth Edition (WISC-V) [21,37]. These criteria are consistent with recommendations for the assessment of twice exceptional children. Then, the students included in the ADHD group were all assessed and diagnosed either by an experienced professional clinician or an intern supervised by an experienced professional clinician. The assessment was based on several sources of information including clinical interviews (e.g., the semi-structured interview K-SADS-PL DSM-5), specific assessments of attention (e.g., the Conners Continuous Performance Test (CPT-3) and the Test of Everyday Attention for Children (TEA-Ch)) and executive functions (e.g., the Delis–Kaplan Executive Function System (D-KEFS) and the Tower of London test), in addition to the use of relevant standardized behavioral rating scales (e.g., the Behavior Assessment System for Children (BASC-3) and the Conners 3 rating scale). Finally, for a child to be included in the gifted/ADHD group, they had to meet the inclusion criteria for both the gifted and ADHD groups.

Students with a mental health disorder (e.g., generalized anxiety disorder and depression) were included in the study to increase the representativeness of the results, thus addressing a limitation raised in previous studies of youth with ADHD [38] or giftedness [39,40]. In contrast, students whose assessment concluded autism spectrum disorder or intellectual disability (in the ADHD group) were excluded to avoid confounders specifically related to these disorders.

### 2.2. Measures

The measures (extracted from archival data) included in the current study were the Wechsler Intelligence Scale for Children (fifth edition; WISC-V) [41] and the Conners Rating Scale (third edition; Conners-3), all of which were answered in French [42].

The Wechsler Intelligence Scale for Children—fifth edition (WISC-V) [41] is the most widely used test of intellectual ability for children aged six to 16 years [43]. It is individually administered and has seven core subtests (i.e., similarities, vocabulary, block design, matrix reasoning, figure weights, digit span, and coding) that contribute to full-scale intelligence (FSIQ). The FISQ has a standardized mean of 100 (SD = 15). Additionally, the General Ability Index (GAI) was calculated because it provides an estimate of general intellectual ability that is less dependent on working memory and processing speed than the FSIQ. This score is derived from five core subtests (i.e., similarities, vocabulary, block design, matrix reasoning, figure weights). Several studies have shown the relevance of using the GAI instead of the FISQ with gifted children who also have co-occurring disabilities such as ADHD who generally present a weakness in working memory and processing speed [44,45,46]. The WISC-V has good reliability and validity across various populations, including the French Canadian population [47,48,49,50]. The WISC-V scores in the present study are based on French Canadian norms.

The Conners Rating Scales—3rd Edition [42] is widely used in clinical and research settings for assessing the cognitive, emotional, and behavioral symptoms of ADHD and comorbid disorders based on the DSM-5 diagnostic criteria [42]. Coupled with clinical expertise and other clinical instruments, the Conners 3 scale may represent a useful tool to support clinicians in the diagnosis of ADHD [15]. The Conners 3 scale can be completed by parents, teachers, and children themselves, which is a strength of the tool as it allows for observations by multiple informants in different contexts [15]. The Conners 3 rating scale requires the respondent (i.e., parents, teachers, or the child him/herself) to indicate the degree or frequency of each behavior described in the item on a scale of 0 (not true at all), 1 (just a little true), 2 (pretty much true), or 3 (very much true) [51,52]. The items describe the cognitive, emotional, and behavioral symptoms of ADHD and comorbid disorders in children and adolescents ranging from six to eighteen years old. The items on the Conners 3—Teacher target the student’s concentration behavior in the classroom. The items on the Conners 3: Parents often overlap with those on the teacher form. Nevertheless, these items target more specifically the attention and emotions experienced at home. The Conners 3 scale has six content scales (i.e., inattention, hyperactivity/impulsivity, learning problems, executive functioning, defiance/aggression, and peer relations) and four DSM-5 symptom scales (i.e., ADHD predominantly inattentive type, ADHD predominantly hyperactive–impulsive type, conduct disorder, and oppositional defiant disorder) included in both parents’ and teachers’ rating forms. Scale scores are derived by summing the responses of the items in respective scales, then transforming these raw scores to age- and gender-normed t scores (M = 50, SD = 10). High scores indicate more severe problems. While a score between 65 and 69 is considered elevated, a score above 70 is considered very elevated. The French version of the Conners 3 has satisfactory internal dimensional consistency and good item reliability [53].

### 2.3. Statistical Analyses

Analyses were conducted using IBM SPSS (Version 27). The data were screened for normality. The method used in handling data is the listwise detection [54]. Thus, the cases with missing data were omitted and the analyses were performed on the remaining data. The MANOVAs were conducted to compare the three study groups in terms of their IQ and age. MANOVAs were also performed to compare parents and teachers’ responses to the Conners 3 behavioral rating scale for gifted/ADHD children and those for gifted and ADHD children, which was the primary objective of this study. Group membership (i.e., gifted/ADHD, ADHD, and gifted) was the independent variable, and the six Conners 3 content scales (i.e., inattentive, hyperactivity–impulsivity, learning problems, executive functioning, aggression, and peer relations), as well as the four DSM-5 symptoms content scale (i.e., ADHD inattentive type, ADHD hyperactive type, conduct disorder, and oppositional disorder), derived from both parents and teachers, were the dependent variables. Because many of the same items constitute the Conners 3 content and symptom scales, two MANOVAs were conducted separately for these two scales to avoid overlapping. Bonferroni corrections were applied to adjust *p*-values because of the increased risk of a type I error when making multiples comparisons. For significant MANOVAs, Bonferroni post hoc tests and discriminant analyses were conducted. Conducting of these two types of analyses followed a recommendation by Field (2013) [55] that a discriminant analysis should follow a significant MANOVA as it allows for a more detailed decomposition of the linear combination. However, as this analysis does not clarify between which groups the differences lie, a Bonferroni post hoc test was also performed.

Repeated-measures ANOVAs were conducted to explore the differences between mothers’, fathers’, and teacher’s responses to the Conners 3 scale. Each scale was a three-level, within-subject factor that considered all three respondents (i.e., mothers, fathers, and teachers), and group membership was the between-subject factor. Following Field’s [55] recommendation, even though the sphericity assumption was not violated, the Greenhouse–Geisser correction was used. In the case of a significant interaction effect, simple effect analyses were performed to explore the effect of the respondent for each of the groups. When a respondent effect was significant for a particular group or for all groups, contrast analyses were conducted to specify between which respondents the difference lies. For MANOVAs and repeated-measures ANOVAs, effect sizes are reported in terms of partial eta squared (η_p_^2^; 0.14 = large, 0.06 = medium, 0.01 = small) [56].

## 3. Results

Table 1 shows the mean (and standard deviation) scores for age and IQ from the FISQ and GAI of participants in the different groups, as well as the results of the group comparisons. Using the Bonferroni post hoc test, the three groups of children differed from each other in terms of IQ measured by the FISQ and the GAI (all *ps* < 0.03–0.001). Additionally, no significant differences were found in terms of age.

### 3.1. Between-Group Comparisons on the Conners 3 Content and Symptom Scales

The MANOVA model addressing the first hypothesis compared the responses to the six content scales and the four DSM-5 symptom scales of the Conners 3 rating scale of the parents and teachers of ADHD children, gifted children, and gifted/ADHD children. Using Wilk’s lambda, there were significant differences in ADHD symptoms at the content scale (overall), λ = 0.12, F(36, 116) = 4.67, *p* < 0.001, η_p_^2^ = 0.70, and the symptoms scale (overall), λ = 0.24, F(24, 132) = 5.75, *p* < 0.001, η_p_^2^ = 0.51. As is shown in Table 2, the ANOVAs revealed a significant effect of group for most content and symptom scales. Non-significant effects of groups were observed in terms of “peer relations” as perceived by the mothers and fathers. ANOVAs also indicated that there were non-significant effects of the group on “conduct disorder”, “oppositional disorder”, and “aggression” according to the teacher.

The Bonferroni post hoc tests following the significant MANOVAs showed that compared to gifted students, the parents and teachers of ADHD and gifted/ADHD students rated them significantly higher on the “inattention”, “ADHD inattentive type”, “hyperactivity-impulsivity", “ADHD hyperactive type”, “learning problems”, and “executive functions” scales (see Table 2). Moreover, mothers and fathers of gifted/ADHD and ADHD children rated them significantly higher on the “conduct disorder” scale than parents of gifted children. The gifted/ADHD and ADHD groups significantly differ in the teacher ratings of “learn-ing problems”, “executive functioning”, and “ADHD hyperactive type” scales. In fact, teachers observed more learning and executive functioning problems in ADHD students than in gifted/ADHD students. However, they observed more ADHD hyperactive symp-toms in gifted/ADHD children than in ADHD children. Additionally, the parents of gift-ed/ADHD children were more likely to highly rate the “aggression” and the “oppositional disorder” scales than those of gifted children. Furthermore, there were significant differ-ences in fathers’ ratings between gifted children and ADHD children on the “aggression” scale. Moreover, mothers and fathers of ADHD children rated them significantly higher on the “oppositional disorder” scale than those of gifted children. Finally, teachers observed significantly more peer relation problems in ADHD children than in gifted children.

**Figure 1:**
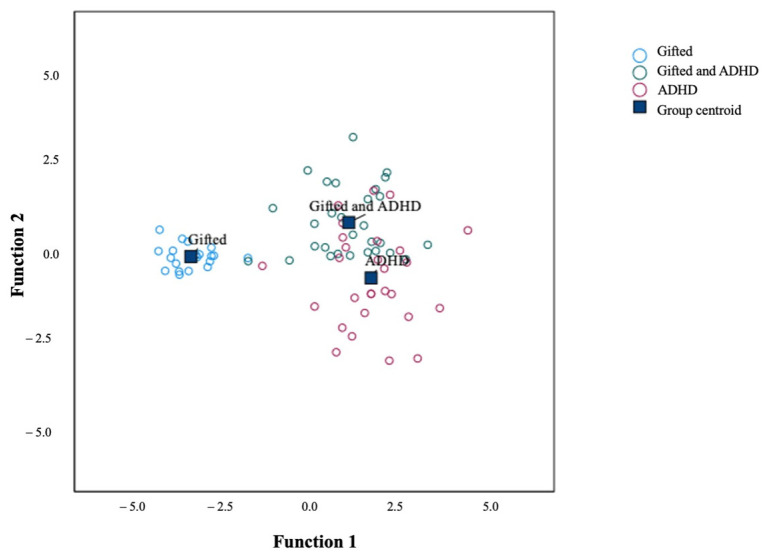
Canonical discriminant function for the Conners 3 content scales.

Significant MANOVAs were also followed by discriminant analysis to supplement the results of the simple group comparison, addressing the second hypothesis. Discriminant analysis was used to classify children into the three study groups on the basis of their ADHD symptoms on the six content scales and the four symptoms’ scales of the Conners 3 scale according to their parents’ and teachers’ perceptions. Regarding the discriminant analysis of content scales, it revealed two discriminant functions. The first explained 90.90% of the variance, canonical R^2^ = 0.82, whereas the second explained only 9.10%, canonical R^2^ = 0.31. In combination, these discriminant functions significantly differentiated the groups of children, λ = 0.12, χ^2^(36) = 139.41, *p* < 0.001, but removing the first function indicated that the second function did not significantly differentiate the groups, λ = 0.69, χ^2^(17) = 25.08, *p* = 0.093. The discriminant function plot showed that the first function discriminated gifted children from gifted/ADHD children and ADHD children, and the second function differentiated gifted/ADHD children from gifted children and ADHD children (see Figure 1). The standardized canonical discriminant function coefficients are presented in Table 3. They indicate that “inattention” according to the mother (r = 0.79) and “learning problems” according to the teacher (r = 0.56) were the variables with the highest load in the first discriminant function. As for the second discriminant function, “hyperactivity–impulsivity” (r = 0.63) and “executive functioning” (r = −0.65) according to the teacher were the variables with the highest load. A total of 78.20% of the original grouped cases were correctly classified. More precisely, 100% of the gifted children were correctly classified, 72.41% of the gifted/ADHD children were correctly classified, and 67.86% of the ADHD children were correctly classified.

**Figure 2:**
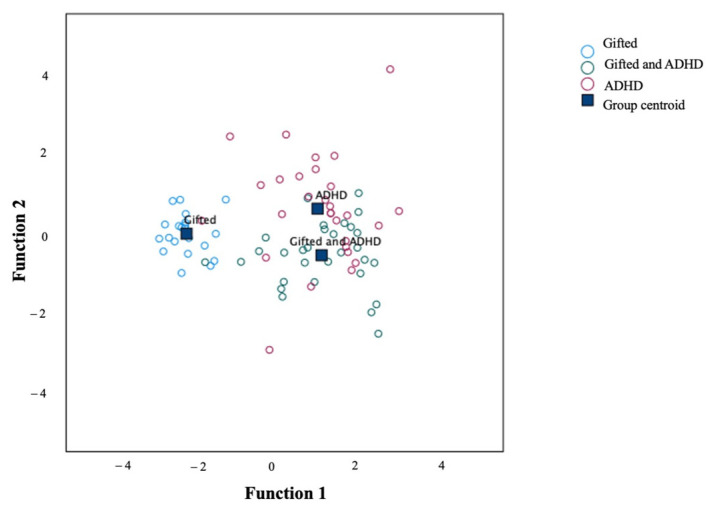
Canonical discriminant functions for the Conners 3 symptom scales.

The discriminant analysis of the symptom’s scales also revealed two discriminant functions. In combination, the first function, explaining 89.60% of the variance (canonical R^2^ = 0.84), and the second function, explaining 10.40% of the variance (canonical R^2^ = 0.46), significantly differentiated between the groups of children, λ = 0.24, χ^2^(24) = 102.34, *p* < 0.001. However, when removing the first function, the second one did not significantly differentiate between the groups, λ = 0.79, χ^2^(11) = 16.94, *p* = 0.110. The discriminant function plot showed that the first function discriminated gifted children from gifted/ADHD children and ADHD children, and the second function discriminated gifted/ADHD children from gifted children and ADHD children (see Figure 2). Standardized coefficients revealed that only “ADHD inattentive type” according to the mother loaded highly onto the first function (r = 0.48). This means that maternal responses to the “ADHD inattentive type” scale contribute the most to the separation of the gifted group. As for the second discriminant function, “conduct disorder” (r = −1.01) and “oppositional disorder” (r = 0.92) according to the father were the variables with the highest load. Additionally, the “ADHD inattentive type” scale according to the teacher was high for both functions (r = 0.67 for the first and r = 0.70 for the second). For the symptom’s scales, 76.30% of the original grouped cases were correctly classified. More precisely, 100% of the gifted children were correctly classified, 70.00% of the gifted/ADHD children were correctly classified, and 65.51% of the ADHD children were correctly classified.

### 3.2. Comparison between Respondents in Their Ratings on the Conners 3 Content and Symptom Scales

The repeated-measures ANOVA design addressing the third hypothesis compared mothers’, fathers’, and teachers’ responses to the Conners 3 content and symptom scales across the three study groups. Table 4 presents the results of the repeated-measures ANOVA. The results showed significant respondent × group interaction effects specific to the “inattention”, “learning problems”, “executive functioning”, “peer relations”, and “conduct disorder” scales. A simple effects analysis was used to decompose the results of the interaction effects. These analyses showed that there was a significant difference in the responses of mothers, fathers, and teachers that were specific to the gifted/ADHD group on the “inattention” scale (F(2, 160) = 15.12, *p* < 0.001), the “learning problems” scale (F(2, 156) = 5.97, *p* = 0.003), the “executive functioning” scale (F(2, 158) = 4.20, *p* = 0.017), and the “peer relations” scale (F(2, 158) = 3.84, *p* = 0.023). These analyses also showed that there was a significant difference in the rating of the respondents that was specific to the ADHD group on the “learning problems” scale (F(2, 156) = 4.69, *p* = 0.010), the “executive functioning” scale (F(2, 158) = 6.71, *p* = 0.002), and the “conduct disorder” scale (F(2, 156) = 7.24, *p* = 0.001). Furthermore, for scales with no respondent × group interaction effects, the results revealed the main effects of respondents for all groups combined.

A contrast analysis was performed by applying the Bonferroni correction (α/3 = 0.017) to establish between which respondents the differences were significant. These analyses showed that teachers tended to rate students in the gifted/ADHD group lower on the “inattention” scale than mothers (F(1, 32) = 15.69, *p* < 0.001, η_p_^2^ = 0.34) and fathers (F(1, 32) = 14.97, *p* < 0.001, η_p_^2^ = 0.32). They also tended to rate these students lower than mothers on the “learning problems” (F(1, 30) = 8.23, *p* = 0.007, η_p_^2^ = 0.22) and the “executive functioning” scales (F(1, 31) = 7.84, *p* = 0.009, η_p_^2^ = 0.20). Additionally, fathers were likely to rate ADHD students less highly on the “executive functioning” scale than mothers (F(1, 28) = 6.68, *p* = 0.015 η_p_^2^ = 0.193) and teachers (F(1, 28) = 9.16, *p* = 0.005, η_p_^2^ = 0.25). As for the mothers of ADHD children, they were more likely to rate them higher on the “conduct disorder” scale than fathers (F(1, 28) = 7.25, *p* = 0.012, η_p_^2^ = 0.21) and teachers (F(1, 28) = 7.10, *p* = 0.013, η_p_^2^ = 0.20).

Table 5 presents the results of the contrast analysis following a significant respondent main effect. Mothers tended to rate students in all groups higher on the “hyperactive–impulsive” scale than teachers. They were also likely to respond more strongly to the “aggression” scale than fathers and teachers. Regarding this scale also, teachers tended to rate students lower than fathers. Additionally, mothers were likely to respond more strongly to the “ADHD inattentive type” scale than fathers. Finally, they also tended to rate students higher on the “ADHD hyperactive type” and the “oppositional disorder” scales than fathers and teachers.

## 4. Discussion

The main objective of this study was to examine parents’ and teachers’ responses to the Conners 3 behavioral rating scale regarding gifted/ADHD children compared to gifted children without ADHD and non-gifted ADHD children. The research findings suggested that there were differences between the three groups for all symptoms assessed by the Conners 3 scale, except for those of aggression, conduct disorder, and oppositional disorder according to the teacher, as well as for those of peer relations according to the mother and the father. Specifically, on the basis of responses from mothers, fathers, and teachers, significant differences between the three groups, supported by large effect sizes, were observed for inattention problems (0.53 < η_p_^2^ < 0.60), executive function problems (0.27 < η_p_^2^ < 0.53), and learning problems (0.30 < η_p_^2^ < 0.47).

Our first hypothesis of research (H1) stating that parents and teachers would report more ADHD symptoms in ADHD children than in gifted children or in gifted/ADHD children was partially confirmed. On the one hand, parents and teachers of ADHD children reported more problems with inattention, learning, and hyperactivity–impulsivity than those of gifted children. On the other hand, although teachers reported more inattention and learning problems in ADHD students than in gifted/ADHD students, they reported more hyperactivity–impulsivity problems (DSM-5 scale) in gifted/ADHD children than in ADHD children. As for the second hypothesis (H2) stating that symptoms of inattention, hyperactivity–impulsivity, and learning problems perceived by the parents and the teacher would significantly distinguish gifted students from gifted/ADHD and ADHD students, it was also partially confirmed. As a matter of fact, results from the first discriminant analysis showed that the distinction between the gifted group and the two other groups was mainly explained by the fact that they had fewer attention problems as perceived by the mothers, as well as fewer learning problems as perceived by the teachers. Thus, symptoms of hyperactivity–impulsivity contributed little to this distinction. However, it should be noted that one the clearest distinction between the gifted/ADHD group and the other two groups from this analysis was that the gifted/ADHD group was perceived by teachers as having more hyperactivity–impulsivity symptoms than the others.

These findings are consistent with those of Gomez and colleagues [34], who found that mothers of gifted children reported fewer symptoms of inattention than those of gifted/ADHD and ADHD children. As Foley-Nicpon [27] suggested, it is possible that the gifted/ADHD children included in our sample were having difficulties compensating for their attentional difficulties with their intelligence, creativity, or talent since the demands of their environment had become too high. In this regard, the average age of children in the gifted/ADHD group is nine years old, which is the age at which children begin the second cycle of elementary school in Quebec. At this level, the learning experience increases in complexity, which demands more attention from the children [57]. In this context, the children’s academic performances may decline, even if their marks remain within the average of the school group [58]. Thus, this decline may be even more noticeable to the children’s mothers who have a comparison of their academic potential with previous school years, which is not necessarily the case for the teachers. Indeed, Brown and colleagues [19] found that gifted/ADHD students were more likely to be placed in special education settings, or to use tutoring services in comparison to gifted peers without ADHD. Thus, parents’ and teachers’ perceptions of the gifted group as having fewer learning problems than the other two groups corroborated several studies indicating that difficulties associated with ADHD represent barriers to learning, which can have a negative impact on academic achievement [59,60,61]. In fact, in their study, Zentall and colleagues found that both gifted/ADHD and ADHD students were generally described as underachievers [62]. This underperformance in school can be explained by the executive function impairment associated with ADHD [19]. To this end, the fact that teachers observed more executive functioning difficulties in ADHD children than in gifted/ADHD children is then consistent with the study of Whitaker and colleagues [63], who found that gifted/ADHD students performed better on an executive function task (e.g., strategic verbal memory) without organizational cues than ADHD students. Thus, it is possible that their intelligence may compensate, to some extent, for their executive functioning impairment, explaining the fact that, according to teachers, they have significantly fewer learning problems than ADHD children.

In terms of symptoms of hyperactivity–impulsivity, the fact that parents and teachers reported more hyperactive–impulsive behaviors in gifted/ADHD and ADHD children than in gifted children is consistent with the study by Gomez and colleagues [34]. Moreover, like these researchers, when only mother ratings were considered, gifted/ADHD and ADHD children showed similar levels of hyperactivity–impulsivity symptoms. However, considering the teachers’ observations—which was not done by Gomez and colleagues [34]—gifted/ADHD children showed more hyperactivity–impulsivity symptoms than ADHD children. In addition to motor activity, it is possible that verbal activity and questioning thinking as hyperactivity–impulsivity, behaviors generally more prominent in gifted/ADHD children, may have distinguished them from ADHD children by their teachers [34]. Furthermore, the fact that the only differences between gifted/ADHD children and ADHD children were observed in terms of hyperactive–impulsive symptoms, learning difficulties, executive functioning by the teachers suggests that their observations, in addition to those of the parents, are very important in the assessment of ADHD in order to limit misdiagnosis in the context of giftedness.

The results revealed that the mothers and fathers of gifted/ADHD and ADHD children observed more aggression and oppositional behavior than those of gifted children. Moreover, while mothers and fathers reported more conduct disorder behavior in gifted/ADHD children than in gifted children, only the mothers of ADHD children reported more of this behavior in their children than in gifted children. This is consistent with the study by Antshel and colleagues [64], which indicated that gifted/ADHD have higher rates of comorbidities than gifted children, similar to those of ADHD children. These findings suggest that even in the presence of giftedness, ADHD is a risk factor for psychosocial adjustment and that it should be investigated in order to better understand the circumstances leading to psychosocial adjustment difficulties in gifted/ADHD youth.

A secondary objective of this study was to explore the differences between mothers’, fathers’, and teachers’ responses to the Conners 3 rating scale. The results showed that in general, mothers reported more ADHD behaviors than fathers and teachers, supporting our third hypothesis (H3). On the one hand, this finding is consistent with many studies that have shown that mothers are more likely to rate their children as having more behavioral problems than fathers [65,66,67,68]. In this regard, Sollie and colleagues [68] noted in their study that the way ADHD symptoms were rated was notably influenced by the gender of the parent. To this end, Climie and Mitchelle [69] observed that emotional frustration represented a predictor of high ADHD behavior ratings for parents. Given that mothers tended to make personal attributions of their child’s negative behaviors (e.g., inattention and hyperactivity–impulsivity) and situational attributions of prosocial behavior [70], it is possible that this tendency had exacerbated their emotional frustration with their child’s ADHD-related behaviors, which is why they rated their child’s behaviors higher on the Conners 3 scale than fathers and teachers. On the other hand, this result is consistent with several studies that have shown that parents reported more behavioral problems in children than teachers regarding ADHD symptoms [68,71,72]. However, it is important to note that other studies have found the opposite [73]. These discrepant results reflect the importance of the context in which a child is observed, which can influence the type and frequency of behaviors reported by different informants [68]. In the context of this study, in which all children visited a psychology or neuropsychology clinic, it is possible that the child’s difficulties may affect the mother to a greater extent than the teacher, hence the need for their consultation, which may have influenced scoring questionnaires such as the Conners 3 scale.

For the gifted/ADHD group, the results revealed that both mothers and fathers tended to report more inattentive behavior in their child than teachers. Specifically, attention difficulties were found to be clinically significant, according to both parents (T-score ≥ 70), while they were found to be high or average according to the teacher. This finding is consistent with that of Wood [31], who observed that teachers rated inattentive behaviors of gifted youth suspected of ADHD as average. Additionally, our results showed that mothers were more likely to observe learning and executive function problems in their child than teachers. All these findings may be related to the concept of the masking effect [14]. On the one hand, since giftedness can mask the difficulties of ADHD, it can be difficult for teachers to notice the telltale signs of ADHD in gifted students [14]. Indeed, the clinical profile of ADHD in these students is generally not as clear-cut as in those with ADHD only [74]. On the other hand, through their role, parents are generally aware of the extent of their child’s abilities in different contexts. Thus, when they see that their child’s potential is not being realized in the school environment to the extent of his or her abilities, it is possible that they are more attentive to the explanatory causes of this [75]. Furthermore, it can be energy-consuming for gifted/ADHD children to constantly compensate for their undiagnosed difficulties with their intellectual abilities during the school day [76]. Thus, once at home, the cognitive fatigue that has accumulated can interfere with their compensation mechanisms. In this context, parents may be able to observe difficulties in attention, executive functions, and learning to a greater extent.

For the group of children with ADHD, the results raised differences among respondents in the way they rated the executive function and conduct disorder behaviors. First, fathers tended to report less executive function problems than teachers and mothers in children with ADHD, which is consistent with the results of previous studies [77,78,79]. However, other studies have found the opposite [80,81]. Once again, these results show the importance of considering the perceptions and expectations of the raters, as well as the context in which the child is observed, knowing that his or her behavior may vary according to the setting [79]. Firstly, the school environment requires the child to use multiple executive functions to accomplish school tasks. Because this environment is more structured and less flexible than the home, it may further highlight the child’s executive function difficulties. Additionally, it is possible that teachers have an easier time identifying executive function deficits in children due to their training and familiarity with age-appropriate behaviors [79]. Thus, given that ADHD students cannot compensate for their executive function difficulties to the same extent as gifted/ADHD students with their intellectual abilities, this may facilitate teachers’ identification of these difficulties in ADHD students. Furthermore, the fact the mothers tended to report more executive function problems than the father may be because they observe them more frequently in settings that require the use of executive functions, such as when doing their homework, than fathers. In Canada in 2015, 65% of the total hours associated with childcare, including homework time, were supervised by women, while 35% of those hours were supervised by men [82]. Finally, mothers also tended to report more oppositional behaviors in their child than fathers and teachers. This finding is consistent with those of Maniadaki and colleagues [83], which indicated that mothers tended to rate conduct problems as being more severe and more negatively impacting their children’s lives than ADHD behaviors. One possible explanation raised by Lewis and colleagues [84] is that mothers of children with oppositional behaviors have difficulty integrating the conflicting views of their child’s behavior, which is associated with strong emotional reactions. Indeed, they could describe them as “manipulative” on the one hand and “vulnerable” on the other. 

## 5. Strengths, Limitations, and Future Directions

First, one of the strengths of the study is that it draws on the perceptions of multiple informants (e.g., fathers, mothers, and teachers), as is suggested in the clinical assessment of ADHD [15]. A second strength is that the assignment of youth to the different groups is based on rigorous ADHD and giftedness assessments by trained clinicians or interns supervised by an experienced clinicians. However, the limitations of this study must be considered. First, the lack of a control group made it impossible to compare gifted and non-gifted children [40]. Additionally, the representativeness of the sample is limited considering that only 25% of the sample were girls. In this regard, the scientific literature indicates that there is an under-identification of giftedness among girls and some children from marginalized groups [85,86,87]. Thus, to generalize the results to all children, future studies would benefit from including more girls, as well as children of different ethnicities and socioeconomic backgrounds. Finally, it is important to consider that the results of the present study are based on analyses that were carried out on a clinical sample. Thus, it is possible to assume that the consultation of the child with his or her parents in a psychology or neuropsychology clinic may be motivated by finding explanatory leads to the distress experienced by the child. This distress may have colored the way parents were invested in the assessment process (e.g., their responses to the Conners 3 scale). In this context, samples for future studies of giftedness should consist of as many children who have been formally identified as gifted as are suspected of being gifted. 

## 6. Conclusions

This study is innovative in that it explored parents’ and teachers’ responses to the Conners 3 behavioral rating scale to better understand the clinical profile of gifted/ADHD youth. The findings suggested that while the use of the Conners 3 scale clearly distinguishes the gifted group from the other two, it does not clearly distinguish the gifted/ADHD group from the ADHD group. The learning difficulties observed by teachers was the most significant element that distinguished gifted/ADHD students from ADHD students. Additionally, the fact that the parents of gifted/ADHD children rated inattentive behaviors more highly than teachers and that mothers rated behaviors related to learning problems and executive functions more highly than teachers highlights the issue of the masking effect surrounding the identification of a dual neurodevelopmental condition. Thus, the results raised the importance of the complementarity of the different informants in the process of the evaluation of ADHD in a context of giftedness to counter the masking effect. Moreover, in a context where the scientific literature raises the risk of misdiagnosis between ADHD and giftedness [14], it would be useful if behavioral rating scales such as the Conners 3 scale included scales targeting certain characteristics of giftedness, as is the case for scales measuring certain disorders comorbid with ADHD, in order to allow for a better differential diagnosis. 

## Figures and Tables

**Table 1:** Mean (and standard deviation) scores for IQ and age in different groups and results of group comparisons.

	Gifted	Gifted/ADHD	ADHD	Group Comparison		
Variables	*M (SD)*	*M (SD)*	*M (SD)*	*F* (*df* = 2)	*p*	ƞ_p_^2^
Age	9.63 (2.41)	9.90 (2.78)	9.95 (2.51)	0.12	0.889	0.003
FISQ	136.55 (7.12)	125.77 (7.22)	104.46 (7.91)	140.07	<0.001	0.76
GAI	138.14 (8.11)	132.26 (8.32)	108.00 (7.99)	118.42	<0.001	0.73

FISQ: full-scale intelligence quotient from the Wechsler Intelligence Scale for Children—fifth edition (WISC-V); GAI: general ability index from the WISC-V; ƞ_p_^2^: partial eta squared.

**Table 2:** Statistical comparison between the average scores of the different groups to the Conners 3.

							Bonferroni Post-Hoc Test		
	Gifted/ADHD (*n* = 32)	Gifted (*n* = 21)	ADHD (*n* = 29)	Group Comparaison			Gifted/ADHD vs. Gifted	Gifted/ADHD vs. ADHD	Gifted vs. ADHD
Variables	*M (SD)*	*M* (*SD*)	*M* (*SD*)	*F* (*df* =2)	*p*	η_p_^2^	p *	p *	p *
Content Scales									
Inattention M	72.41 (10.94)	46.38 (4.65)	70.71 (9.96)	56.77	<0.001	0.60	<0.001	1.000	<0.001
Inattention F	72.35 (9.45)	45.23 (3.30)	66.39 (13.63)	45.95	<0.001	0.55	<0.001	0.090	<0.001
Inattention T	63.66 (11.70)	44.86 (2.63)	67.67 (8.98)	41.80	<0.001	0.53	<0.001	0.293	<0.001
Hyperactivity–Impusivity M	64.63 (15.98)	51.57 (6.04)	62.93 (13.83)	6.70	0.002	0.15	0.003	1.000	0.012
Hyperactivity–Impusivity F	63.14 (15.95)	48.00 (5.93)	60.64 (15.05)	8.22	<0.001	0.18	<0.001	1.000	0.006
Hyperactivity–Impusivity T	60.76 (15.28)	46.81 (3.64)	57.68 (14.31)	7.63	<0.001	0.17	<0.001	1.000	0.013
Learning problems M	61.55 (12.50)	45.29 (3.68)	64.21 (12.80)	20.01	<0.001	0.35	<0.001	1.000	<0.001
Learning problems F	61.28 (12.38)	43.95 (5.77)	58.64 (13.08)	15.82	<0.001	0.30	<0.001	1.000	<0.001
Learning problems T	55.14 (10.82)	41.38 (1.28)	65.75 (13.16)	33.45	<0.001	0.47	<0.001	<0.001	<0.001
Executive functioning M	62.45 (11.93)	46.33 (6.16)	62.82 (10.79)	19.38	<0.001	0.34	<0.001	1.000	<0.001
Executive functioning F	60.41 (12.43)	44.48 (7.14)	56.43 (11.09)	13.95	<0.001	0.27	<0.001	0.498	<0.001
Executive functioning T	58.10 (9.61)	41.29 (2.83)	63.82 (10.45)	42.42	<0.001	0.53	<0.001	0.045	<0.001
Agression M	64.79 (15.61)	51.86 (5.43)	60.89 (15.79)	5.52	0.006	0.13	0.005	0.862	0.076
Agression F	61.28 (17.25)	48.19 (5.58)	58.82 (15.36)	5.51	0.006	0.13	0.006	1.000	0.036
Agression T	53.55 (13.37)	47.76 (5.08)	52.21 (11.98)	1.73	0.184	0.04	0.222	1.000	0.512
Peer relations M	55.65 (12.74)	53.14 (7.12)	51.89 (11.88)	0.83	0.438	0.02	1.000	0.623	1.000
Peer relations F	56.90 (16.18)	48.76 (9.13)	53.39 (13.64)	2.16	0.120	0.05	0.124	1.000	0.733
Peer relations T	50.86 (9.03)	47.81 (5.04)	57.46 (15.84)	4.82	0.011	0.11	1.000	0.091	0.012
DSM-5 Symptoms Scales									
ADHD inattentive type M	71.63 (11.41)	46.38 (5.47)	69.03 (11.45)	43.07	<0.001	0.53	<0.001	0.996	<0.001
ADHD inattentive type F	69.30 (10.96)	45.53 (4.19)	63.38 (12.58)	33.81	<0.001	0.47	<0.001	0.094	<0.001
ADHD inattentive type T	66.10 (11.56)	42.52 (2.87)	68.38 (10.24)	52.53	<0.001	0.58	<0.001	1.000	<0.001
ADHD hyperactive type M	63.50 (15.27)	51.43 (6.71)	61.72 (13.07)	6.15	0.003	0.14	0.004	1.000	0.018
ADHD hyperactive type F	61.80 (15.18)	46.29 (6.14)	58.35 (15.45)	8.54	<0.001	0.18	<0.001	0.991	0.008
ADHD hyperactive type T	61.87 (15.92)	46.90 (3.16)	57.17 (14.40)	8.08	<0.001	0.17	<0.001	0.024	0.012
Conduct disorder M	53.10 (10.22)	45.00 (4.01)	55.59 (15.26)	5.60	0.005	0.13	0.042	1.000	0.005
Conduct disorder F	53.00 (11.88)	44.57 (3.28)	50.17 (9.85)	4.85	0.010	0.11	0.008	0.778	0.132
Conduct disorder T	49.20 (9.38)	46.90 (3.67)	48.28 (7.52)	0.57	0.568	0.02	0.868	1.000	1.000
Oppositional disorder M	65.60 (15.83)	50.91 (7.69)	64.65 (15.35)	8.06	<0.001	0.17	0.001	1.000	0.003
Oppositional disorder F	61.23 (16.64)	48.00 (6.40)	59.97 (14.42)	6.53	0.002	0.15	0.004	1.000	0.010
Oppositional disorder T	56.50 (14.31)	49.09 (6.95)	55.90 (16.81)	2.08	0.132	0.05	0.183	1.000	0.274

Note. M: mothers; F: fathers; T: teachers; *M*: mean; *SD*: standard deviation; ƞ_p_*^2^*: partial eta squared; * Bonferroni-corrected *p*-values.

**Table 3:** Standardized canonical discriminant function coefficients for the Conners 3 content and the symptoms scales.

Variables	Function 1	Function 2
Content scales		
Inattention M	0.79	−0.04
Inattention F	0.48	0.48
Inattention T	0.24	0.21
Hyperactivity–impulsivity M	0.38	−0.28
Hyperactivity–impulsivity F	−0.04	−0.52
Hyperactivity–impulsivity T	−0.25	0.63
Learning problems M	0.38	−0.28
Learning problems F	−0.21	0.49
Learning problems T	0.56	−0.30
Executive functioning M	−0.48	0.32
Executive functioning F	−0.37	0.18
Executive functioning T	0.15	−0.65
Agression M	0.14	0.26
Agression F	−0.01	0.01
Agression T	0.18	−0.11
Peer relations M	−0.41	0.22
Peer relations F	0.45	−0.08
Peer relations T	0.04	−0.44
DSM-5 symptoms scales		
ADHD inattentive type M	0.48	−0.12
ADHD inattentive type F	0.15	−0.86
ADHD inattentive type T	0.67	0.70
ADHD hyperactive type M	−0.04	0.53
ADHD hyperactive type F	0.14	−0.12
ADHD hyperactive type T	0.11	−0.43
Conduct disorder M	0.00	0.66
Conduct disorder F	−0.09	−1.01
Conduct disorder T	0.06	−0.05
Oppositional disorder M	0.01	−0.48
Oppositional disorder F	0.01	0.92
Oppositional disorder T	0.07	0.26

Note. M = mother; F = father; T = teacher.

**Table 4:** Repeated-measures ANOVA statistics for the Conners 3 content and symptoms scales.

	Groups			Respondents			G X R		
Variables	*F* Ratio	*df*	η_p_^2^	*F* Ratio	*df*	η_p_^2^	*F* Ratio	*df*	η_p_^2^
Content scales									
Inattention	100.86 ***	2	0.72	7.39 ***	1.903	0.85	3.90 **	3.806	0.09
Hyperactivity–impulsivity	9.69 ***	2	0.20	6.03 **	1.928	0.07	0.22	3.856	0.01
Learning problems	33.64 ***	2	0.46	3.25 *	1.891	0.04	4.18 **	3.782	0.10
Executive functioning	35.99 ***	2	0.48	4.62 *	1.997	0.06	4.16 **	3.994	0.10
Aggression	7.16 **	2	0.15	12.74 ***	1.805	0.14	0.97	3.610	0.02
Peer relations	1.76	2	0.04	0.66	1.743	0.01	3.82 **	3.485	0.09
Symptoms scales									
ADHD inattentive type	81.83 ***	2	0.67	3.92 *	1.918	0.05	2.32	3.836	0.06
ADHD hyperactive type	10.13 ***	2	0.21	4.10 *	1.837	0.05	0.38	3.674	0.01
Conduct disorder	5.30 **	2	0.12	3.39 *	1.801	0.04	2.92 *	3.602	0.07
Oppositional disorder	7.76 ***	2	0.16	7.68 **	1.587	0.09	0.96	3.173	0.02

Note. G: groups; R: respondents; Greenhouse–Geisser correction was used across the respondents and within subjects. η_p_^2^ = partial eta squared. * *p* < 0.05. ** *p* < 0.01. *** *p* < 0.001.

**Table 5:** Comparison of respondents to the Conners 3 content and symptom scales for all groups combined.

	Mothers vs. Fathers			Mothers vs. Teachers			Fathers vs. Teachers		
Variables	*F* (*df* = 1)	*p*	η_p_^2^	*F* (*df* = 1)	*p*	η_p_^2^	*F* (*df* = 1)	*p*	η_p_^2^
Inattention	2.23	0.076	0.04	14.09 **	<0.001	0.15	4.24	0.043	0.05
Hyperactivity–impulsivity	3.29	0.074	0.04	11.91 **	<0.001	0.13	2.86	0.095	0.04
Learning problems	4.73	0.033	0.06	5.16	0.026	0.06	0.25	0.619	0.00
Executive functioning	6.87 *	0.011	0.08	7.21 *	0.009	0.08	0.00	0.998	0.00
Aggression	7.55 *	0.007	0.09	23.21 **	<0.001	0.23	6.27 *	0.014	0.07
ADHD inattentive type	8.07 *	0.013	0.09	6.14	0.018	0.07	0.02	0.091	0.00
ADHD hyperactive type	3.84 *	0.006	0.05	5.21 *	0.015	0.06	0.85	0.901	0.01
Conduct disorder	3.84	0.054	0.05	5.21	0.025	0.06	0.85	0.360	0.01
Oppositional disorder	11.45 *	0.001	0.13	11.82 **	<0.001	0.13	1.88	0.175	0.02

Note. The contrast analyses were performed on the scales for which a significant main effect of the respondent was observed. η_p_^2^ = partial eta squared. * *p adjusted* < (α of 0.05/3 = 0.017); ** *p* < 0.001.

## Data Availability

The data presented in this study are available upon reasonable request from the corresponding author.
